# Combination of Plant Metabolic Modules Yields Synthetic Synergies

**DOI:** 10.1371/journal.pone.0169778

**Published:** 2017-01-12

**Authors:** Fatemeh Rajabi, Ernst Heene, Jan Maisch, Peter Nick

**Affiliations:** Molecular Cell Biology, Botanical Institute, Karlsruhe Institute of Technology, Germany; Institute of medical research and medicinal plant studies, CAMEROON

## Abstract

The great potential of pharmacologically active secondary plant metabolites is often limited by low yield and availability of the producing plant. Chemical synthesis of these complex compounds is often too expensive. Plant cell fermentation offers an alternative strategy to overcome these limitations. However, production in batch cell cultures remains often inefficient. One reason might be the fact that different cell types have to interact for metabolite maturation, which is poorly mimicked in suspension cell lines. Using alkaloid metabolism of tobacco, we explore an alternative strategy, where the metabolic interactions of different cell types in a plant tissue are technically mimicked based on different plant-cell based metabolic modules. In this study, we simulate the interaction found between the nicotine secreting cells of the root and the nicotine-converting cells of the senescent leaf, generating the target compound nornicotine in the model cell line tobacco BY-2. When the nicotine demethylase *Ntom*CYP82E4 was overexpressed in tobacco BY-2 cells, nornicotine synthesis was triggered, but only to a minor extent. However, we show here that we can improve the production of nornicotine in this cell line by feeding the precursor, nicotine. Engineering of another cell line overexpressing the key enzyme *Ntab*MPO1 allows to stimulate accumulation and secretion of this precursor. We show that the nornicotine production of *Ntom*CYP82E4 cells can be significantly stimulated by feeding conditioned medium from *Ntab*MPO1 overexpressors without any negative effect on the physiology of the cells. Co-cultivation of *Ntom*CYP82E4 with *Ntab*MPO1 stimulated nornicotine accumulation even further, demonstrating that the physical presence of cells was superior to just feeding the conditioned medium collected from the same cells. These results provide a proof of concept that combination of different metabolic modules can improve the productivity for target compounds in plant cell fermentation.

## Introduction

Plants are able to produce a wide variety of specific secondary metabolites, which makes them unique among multicellular organisms [1], [2]. Starting from traditional medical systems, humans have exploited this metabolic proficiency of plants since ancient times. Due to the growth of the world population and the popularity of phytomedical compounds, the global demand for natural compounds has been growing steadily, and pharmaceutically interesting plants are increasingly exploited on an industrial scale. However, low yield [3], [4] limited seasonal availability, low abundance of active compounds, and slow growth of many medical plant species [5], [6] are progressively hampering this approach. In addition, in some countries, cultivated or collected medicinal herbs have raised concern due to pollution by herbicides, insecticides, and heavy metals [7]. Moreover, especially in taxonomically difficult groups, or in plants that are rare, adulteration by surrogate material of similar morphology has become a serious issue [8]. Chemical synthesis does not provide alternatives, because due to the complex structures of secondary metabolites, production *in vitro* is not cost-efficient in most cases. Thus, innovative strategies to produce medicinal natural products in sufficient quantity, quality, and standardized conditions have considerable international impact for the development of novel pharmaceutical products [7].

Plant cell cultures certainly represent a valid alternative for the sustainable production of valuable secondary metabolites, but the success of plant cell fermentation has been limited by low product yields and cell culture variability [6]. One reason for this limitation is the fact that, in contrast to the situation in a plant, the metabolic activity in a plant cell culture system is not partitioned to different cell types. In other words, plant cell fermentation in batch cell cultures might often not be very efficient, because it is based on just one type of cells and therefore cannot provide the interaction of different cell types required for the maturation of the metabolites. Synthesis of the alkaloid nornicotine provides a striking example of metabolic partitioning in plants:

Nornicotine synthesis occurs predominantly in leaves, whereas the nornicotine precursor, nicotine, is synthesised in the roots and subsequently transported via the xylem to leaves and the other aerial parts of the plant [9], [10], [11]. Nicotine is primarily stored in the vacuole of the cells and acts as defence mechanism against herbivores [12], [13]. In a final step, the nicotine imported into the leaf is demethylated to nornicotine through an oxidative process catalysed by an enzyme belonging to the cytochrome P_450_ family of monooxygenases [14], [15], [16]. Different from other alkaloid biosynthesis enzymes, nicotine *N*-demethylase (NND) occurs exclusively in leaf tissue rather than in the root, with high levels of activity during leaf senescence [13].

**Fig 1:**
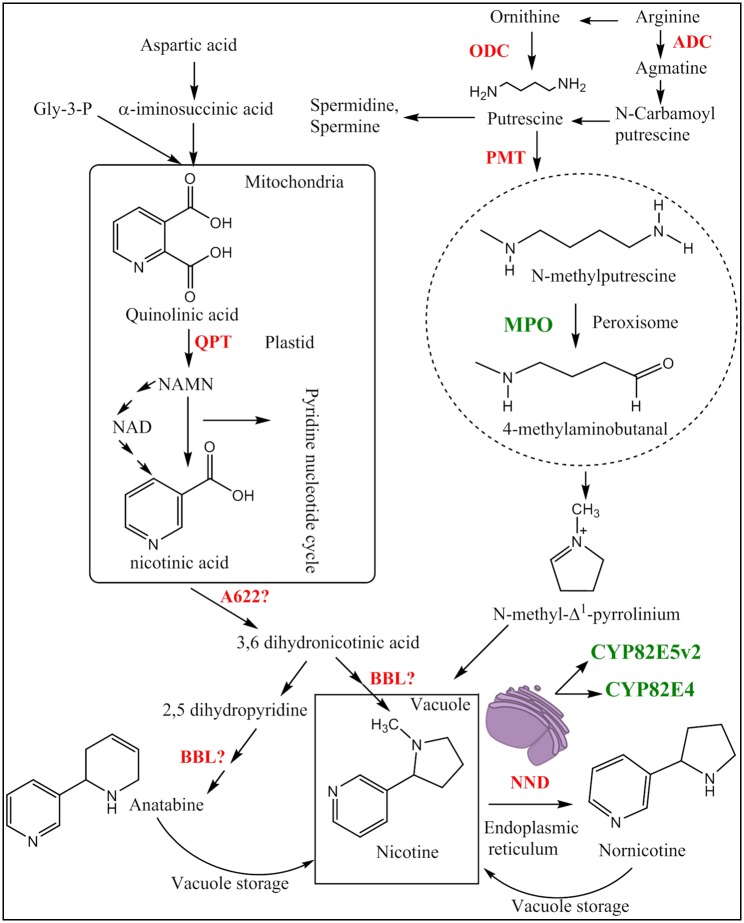
Subcellular compartmentalisation of nicotine / nornicotine biosynthetic enzymes—modified from [13]. A622: isoflavone reductase-like protein; ADC: arginine decarboxylase; BBL: berberine bridge enzyme-like; MPO: N-methylputrescine oxidase; NND: nicotine *N*-demethylase; ODC: ornithine decarboxylase; PMT: putrescine methyltransferase; QPT: quinolinate phosphoribosyltransferase. Genes that have been overexpressed in the present study are shown in green.

This pronounced metabolic partitioning on the level of different cell types is accompanied by a prominent compartmentalisation of different metabolic steps within the cell (Fig 1): The pyrrolidine moiety of nicotine derives from the symmetric diamine putrescine. *N*-methylation of putrescine by putrescine N-methyltransferase (PMT) produces N-methylputrescine. This product is then deaminated oxidatively by N-methylputrescine oxidase (MPO) to form 4-methylaminobutanal. Spontaneous cyclisation of 4-methylaminobutanal produces N-methylpyrrolinium cation (*N*-methyl-Δ^1^-pyrrolinium). MPO belongs to a subclass of diamine oxidases which are dependent on copper and topaquinone for activity and was found to colocalise with the peroxisome in *Nicotiana benthamiana* leaf epidermal cells [17], [18], [19]. Nicotine is then formed through coupling of N-methylpyrrolinium cation and a non-identified intermediate from nicotinic acid [9]. Nicotinic acid formation is through the salvage pathway of nicotinamide adenine dinucleotide (NAD), which is derived from aspartic acid [20]. This aspartate derived pathway is coupled to the pyridine nucleotide cycle by quinolinate phosphoribosyltransferase (QPT). Quinolinate-dependent synthesis of nicotinic acid mononucleotide (NAMN) apparently can occur in both, cytosolic and mitochondrial, compartments [21], [22]. There is good evidence that QPT is targeted to plastids as well [20]. Subsequently, the nicotinic acid precursor is thought to be first reduced, then decarboxylated and eventually coupled to the N-methyl-Δ^1^- pyrrolinium substrate by an unknown mechanism. However, the possible involvement of additional intermediates in these reactions has remained ambiguous. Recent studies indicate a possible involvement of two further gene products (A622 and BBL) during the final stages of nicotine biosynthesis. Although the N-terminal region of BBL enzymes contains putative vacuolar sorting determinants [23], which qualifies these enzymes as nicotine synthases, the molecular nature of the final enzyme and its corresponding pyridine substrate are still unclear [23], [24], [25].

We used nornicotine as a case study to demonstrate the impact of metabolic partitioning. This nicotine metabolite has acquired great interest because of its efficacy against the Alzheimer disease [26]. The nitrosylated product of nornicotine and other main alkaloids of tobacco are considered as potent carcinogens. However these problematic derivatives are mostly formed during the tobacco curing process [27]. Our basic strategy was to experimentally mimic the situation in a plant tissue by coupling two different cell types: the supernatant generated by a donor cell type is added to a metabolically different receiver cell type. Since the biosynthetic pathway for nornicotine is relatively well understood, and most of the enzymes involved in the production of this metabolite are already known [13], it was possible to generate two different metabolic modules based on genetic engineering of the alkaloid biosynthetic pathway in the BY-2 model. Since nicotine alkaloids synthesis is functionally linked with the response to herbivores, it is induced by wounding. Wound signalling is mainly conveyed by jasmonic acid. Therefore, jasmonic acid can be used as an elicitor to activate nicotine alkaloids synthesis [1], [9], [12], [25]. We demonstrate further, how modular combination of these transgenic cell strains yields synergy that cannot be achieved by each of the two cell lines alone.

## Materials and Methods

### Plant material, cell culture and elicitation

Plants of *Nicotiana tabacum* cv 'Bright Yellow 2' (voucher KIT 8579), *Nicotiana paniculata* (voucher KIT 0056), *Nicotiana rustica* (voucher KIT 6534), and *Nicotiana tomentosiformis* (voucher KIT 1367) were provided by the Botanical Garden of the Karlsruhe Institute of Technology. Leaves from fully developed plants were collected from these accessions to determine the content of specific alkaloids. The cell strain BY-2 (*Nicotiana tabacum* L. cv Bright Yellow 2, [28] was cultivated in liquid medium containing 4.3 g^.^L^−1^ Murashige and Skoog salts (Duchefa, http://www.duchefa.com), 30 g^.^L^−1^ sucrose, 200 mg^.^L^−1^ KH_2_PO_4_, 100 mg^.^L^−1^ inositol, 1 mg^.^L^−1^ thiamine, and 0.2 mg^.^L^−1^ (0.9 μM) 2,4-D, pH 5.8. The cells were subcultivated weekly, inoculating 1.0–1.5 mL of stationary cells into fresh medium (30 mL) in 100 mL Erlenmeyer flasks. The cells were incubated at 26°C under constant shaking on a KS260 basic orbital shaker (IKA Labortechnik, http://www.ika.de) at 150 rpm. Every three weeks, stock calli were subcultured on media solidified with 0.8% (w/v) agar (Roth, http://www.carlroth.com). Suspension cultures and calli of the transgenic strains *Ntab*MPO1ox, *Ntab*CYP82E5*v*2ox and *Ntom*CYP82E4ox were cultivated on the same media as non-transformed wild-type cultures (BY-2 WT), but supplemented with 50 mg^.^L^−1^ kanamycin. Jasmonic acid dissolved in ethanol (OlChemIm, Czech Republic) was used as elicitor and was added to the culture medium to a final concentration of 10 μM or 100 μM. Addition of an equivalent volume of ethanol alone served as solvent control. If not stated otherwise, the samples for alkaloid analysis were collected 3 days after subcultivation.

### Generation of GFP-MPO1 and GFP-CYP constructs

2 millimiters of cycling BY-2 WT (3 d after subcultivation, 100 mg of cells) were pipetted onto filter paper to remove the liquid medium. The cells were transferred with a spatula into a 2 mL reaction tube, immediately frozen in liquid nitrogen, and ground with a 5 mm steel bead in a TissueLyser (Qiagen, http://www.qiagen.com). Total RNA was extracted using an RNeasy Plant Mini Kit (Sigma, http://www.sigmaaldrich.com). Optional on-column digestion of genomic DNA was performed with RNase free DNAse I (Sigma) according to the manufacturer instruction. Purity and integrity of the RNA preparation were checked by electrophoresis. RNA was transcribed into cDNA using the SuperScript^®^ II Reverse Transcriptase (https://www.lifetechnologies.com) with 100 ng of RNA as template.

Plasmids for stable and transient transformation of BY-2 WT cells were constructed via Gateway^®^-Cloning (Invitrogen, http://www.invitrogen.com). The cDNA transcripts encoding *Ntab*MPO1 [17], *Ntab*CYP82E5*v*2 [29] and *Ntom*CYP82E4 [30], respectively, were amplified by PCR using appropriate oligonucleotide primers (*Ntab*MPO, 5՛-ATGGCCACTACTAAACAGAAAGTG-3՛ and 5՛-TCAAAGCTTGGCCAGCAAGC-3՛; both *Ntab*CYP82E5*v*2 and *Ntom*CYP82E4, 5՛-ATGCTTTCTCCCATAGAAGC-3՛ and 5՛-TTAATAAAGCTCAGGTGCCAG-3՛). The size of the amplicon was verified by electrophoresis, and amplicons were purified via the NucleoSpin^®^ Extract II kit (Machery-Nagel, http://www.mn-net.com) according to the manufacturer’s instructions. The resulting full-length cDNA of *Ntab*MPO1 was inserted into the binary vector pK7WGF2 producing a fusion, where GFP is N-terminal. For *Ntab*CYP82E5*v*2 and *Ntom*CYP82E4, the binary vector pK7FWG2 [31] was used yielding a fusion, where GFP is C-terminal. All inserts were under control of the constitutive CaMV 35S promoter. The sequence of the fusion construct was verified by restriction digest and sequencing (GATC, http://www.gatc-biotech.com).

### Transient and stable transformation of tobacco BY-2 cells and localisation study

Biolistic transformation was performed as described in [32]. Following bombardment, the cells were incubated for 4–24 h in the dark at 26°C and observed under the fluorescence microscope. Stable transformation of BY-2 cells with the binary vector constructs pK7WGF2-*Ntab*MPO1, pK7FWG2-*Ntab*CYP82E5*v*2, or pK7FWG2-*Ntom*CYP82E4 was achieved based on a modified *Agrobacterium*-based protocol [33]. Cell suspension cultures were established from calli using 50 mg L^−1^ kanamycin added to the liquid medium for selection.

For localisation study of *Ntab*MPO1, the peroxisomal mCherry marker PTS1-mCherry [34] was transiently introduced by particle bombardment into the background of the *Ntab*MPO1-GFP ox line expressing the enzyme as fusion with GFP. Localisation study of *Ntab*CYP82E5*v*2 and *Ntom*CYP82E4 was performed by staining of related overexpressing cell lines with 1 μM ER-Tracker^™^ Red (glibenclamide BODIPY^®^ TR) from Thermo Fisher Scientific according to the manufacturer’s instructions.

### Precursor feeding, combination and co-cultivation experiments

The transformed and non-transformed BY-2 cell suspension cultures were fed with the nornicotine precursor nicotine (Sigma-Aldrich, Munich, Germany). Nicotine was added to the culture medium at a final concentration of 15 ng^.^mL^−1^. For combination experiments, medium from *Ntab*MPO1ox or WT cell lines was collected at day 3 after subcultivation, when cells had reached the maximal proliferation activity. This conditioned supernatant (around 25 ml) was mixed with the same volume of fresh medium and 1.5 ml of stationary *Ntom*CYP82E4 overexpressor cells were then inoculated into 30 ml of this mixture in the same way as during standard subcultivation. The conditioned medium was separated from the cells by sterile filtration with autoclaved nalgene devices (Nalgene, http://www.nalgene.com) using a nylon mesh of 40 μm pore width [35]. For the co-cultivation experiment, we used an inoculum of 3 ml consisting of 50% from *Ntab*MPO1 and 50% of *Ntom*CYP82E4 cells in 60 ml of fresh medium. After 3 days of culture, both cells and medium were analysed for the abundance of alkaloids.

### Determination of transcript abundance

Abundance of CYPs transcripts was quantified by real-time qPCR analysis. Quality and integrity of extracted RNA were analysed using spectrophotometry and agarose gel electrophoresis. First-strand cDNA synthesis was carried out from 1 μg total RNA as described above. Primers for real time PCR were designed using the Primer3 software (http://primer3.ut.ee/). The specificity of the amplification was verified by melting curve analysis and gel electrophoresis, efficiency was determined by analysis of serial cDNA dilution curves. qPCR analysis was carried out in 20 μL reactions containing in final concentration 200 nM of each primer, 200 nM of each dNTP, 1X GoTaq colorless buffer, 2.5 mM MgCl_2_, 0.5 U GoTaq polymerase (Promega, Mannheim, Germany), 1x SYBR green I (Invitrogen, Darmstadt, Germany), and 1 μl of a cDNA template diluted tenfold [36]. Each experiment was repeated in three biological replicates, and the mean fold change was calculated and plotted along with corresponding standard deviation values. The cycling conditions comprised 3 min polymerase activation at 95°C, followed by 40 cycles of strand separation at 95°C for 15 s, annealing and synthesis at 60°C for 40 s. Each assay was performed in triplicate. The relative expression of each gene was calculated with the delta delta C_t_ method [37] using L25 ribosomal protein and elongation factor 1α (EF-1 α) as endogenous control for normalization [38].

### Alkaloid extraction

Nicotine alkaloids were extracted according to techniques developed by Häkkinen and his colleagues [39] with some modifications: To extract tobacco alkaloids, one gram fresh weight of BY-2 cells were dispersed in 2 ml of water. The mixture was basified with 3 ml 3.3% NH_4_OH. To release cell content, cells were lysed by ultrasonication for 2 minutes by means of a high-efficiency ultrasound device (UP 100H, Hielscher, Teltow, Germany) pulsed with 0.5 s intervals using amplitude of 100%. The lysate was spun down for 15 min at 2100 ×g (Z 383 K, Hermle KG, https://www.hermle.de), and the supernatant was collected and extracted with 10 ml dichloromethane. The mixture was incubated for 30 min at ambient temperature on an orbital shaker (150 rpm). Subsequently, the polar dichloromethane layer was separated and collected through a 50-ml separation funnel. In order to improve the efficiency of extraction, this step was repeated. Precipitated proteins were separated by centrifugation from the collected polar phase at 2100 ×g for 15 min. In the next step, the clear lower phase was concentrated by a rotary evaporator (Büchi^®^ rotary evaporator Model R-205, http://www.buchi.com) under a reduced pressure of 550 mbar and a temperature of 40°C. After complete evaporation of dichloromethane, the extract was dissolved in 500 μl of 80% (v/v) methanol for HPLC analysis.

### Separation of alkaloids by High-Performance Liquid Chromatography

The Agilent-1200-Series HPLC system equipped with a diode array detector (G1315D), Agilent ChemStation software and a Phenomenex Gemini-NX 5μ C18 110A 150 mm x 4.6 mm column (Phenomenex, Aschaffenburg, Germany) was used at a column temperature of 35°C and a flow rate of 1.0 ml/min. The injection volume was 20 μl for cell extracts and 30 μl for medium extracts, peaks were quantified at 260 nm. UV spectra were collected over the wavelength range from 200 nm to 700 nm. Eluent A contained 10% acetonitrile in 20 mM ammonium formate adjusted to pH 8.7, and eluent B consisted of 100% acetonitrile. A gradient program was employed composed of a sequence of linear gradients with an initial step of 100% A to 80% A and 20% B over the first 10 min, followed by a second gradient to 10% A and 90% B over the next 10 min, and a final step to 100% B from 21 min after injection till the end of the run at 30 min after injection [40]. The reference alkaloids nicotine, anatabine, anatalline and nornicotine were purchased from Sigma aldrich (Munich, Germany), and used for sample spiking to verify the identified peaks (S1 Fig).

### Phenotyping of transgenic cell lines

The mitotic index (MI) of tobacco BY-2 cell suspension cells was determined following fixation with Carnoy fixative and staining with the nuclear dye Höchst 33258 (2'-(4-hydroxyphenyl)5-(4-methyl-1-piperazinyl)-2,5'-bi(1H-benzimidazole)-trihydrochloride (Sigma–Aldrich, http://www.sigmaaldrich.com), as described in Maisch and Nick (2007) [41]. Cells were observed and captured using an AxioImager Z1 microscope (Zeiss, Jena, Germany). The images were analysed using the AxioVision (Rel. 4.8.2) software (Zeiss, Jena, Germany). To gain more details of the transformed cells, an Axio Observer Z1 microscope (Zeiss, Jena, Germany) in combination with a 63 × /1.44 DIC oil objective and the 488 nm and 561 nm emission lines of the Ar-Kr laser as well as a spinning-disc device (YOKOGAWA CSU-X1 5000) was used. MIs were determined as the relative frequency of mitotic cells out of a sample of 500 cells scored for each data point. Cell length and width were determined from the central section of the cells using the length function of the AxioVision software according to [41]. Each data point represents average and standard error from 500 individual cells from three independent experimental series. Cell viability was analysed by the Evans Blue dye exclusion test [42]. Aliquots (0.5 mL) from each sample were stained with 0.4% (w/v) Evans Blue solution (Sigma-Aldrich) at a ratio of 1:100 (v/v). After incubation for 3 min, the frequency of the unstained (viable) cells was determined as well as the cell number per mL using a hematocytometer (Fuchs-Rosenthal) under bright-field illumination. For each individual sample, 1000 cells were scored. Division synchrony, MI, cell length and width were observed to be unaffected by kanamycin selection as verified by comparison with negative controls cultivated in the absence of the antibiotics. The average length of the cell cycle was estimated from the time course of cell density estimated by a hematocytometer (Fuchs-Rosenthal), using an exponential model for proliferation (N_t_ = N_0_^.^e^kt^ with N_t_ cell density at time point t, N_0_ cell density at inoculation, and k the time constant). In order to set the reference, the starting number (N_0_) was quantified just after subcultivation.

## Results

### Definition of molecular targets for stimulated nornicotine synthesis

In order to design a strategy for metabolic engineering of nornicotine, we focused on MPO1 and CYP82E as rate-limiting enzymes determining the production of nicotine and nornicotine, respectively (Fig 1). Nicotine biosynthesis requires an oxidative deamination of N-methylputrescine, catalyzed by N-methyl putrescine oxidase (MPO1) [GenBank: AB289456.1] [17]. The conversion of nicotine to nornicotine involves *N*-demethylation of nicotine, catalyzed by different isotypes of a cytochrome P_450_ enzyme [15], [29], [43]. Two alleles of this gene, CYP82E, were tested: *Ntab*CYP82E5*v*2 [GenBank: EU182719.1] is found in green leaves of *N*. *tabacum*, a species with low nornicotine levels, whereas *Ntom*CYP82E4 [GenBank: EF042307.1] originates from *Nicotiana tomentosiformis*, a strong converter of nicotine to nornicotine [13], [29].

### Overexpression of MPO1 promotes nicotine accumulation, but does not lead to nornicotine

**Fig 2:**
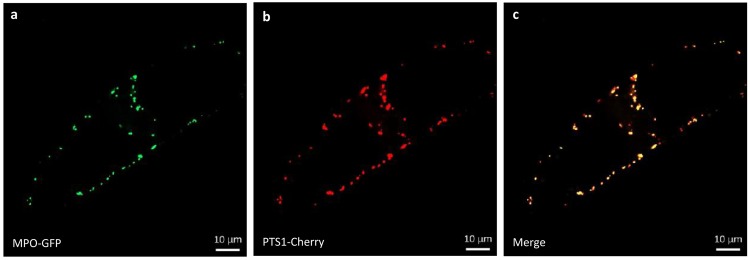
Colocalisation of *Ntab*MPO1-GFP and of PTS1-mCherry in BY-2 cells overexpressing *Ntab*MPO1-GFP showed peroxisome localisation of *Ntab*MPO1. *Ntab*MPO-GFP fluorescence (a) PTS1-mCherry fluorescence (b) and merged fluorescence of a and b (c) Orange colour indicates areas where the images overlap and where the two markers colocalise (Bars = 10 μm).

Since low expression of MPO1 is the main reason for the deficiency of tobacco BY-2 cells to synthesise nicotine efficiently, overexpression of MPO1 represents a strategy to boost nicotine formation [25]. To stimulate the production of nicotine, the native tobacco BY-2 MPO1 cDNA was expressed in fusion with GFP as reporter under the control of the cauliflower mosaic virus (CaMV) 35S promoter in tobacco BY-2 cells using transformation via *Agrobacterium tumefaciens* using kanamycin as selection marker. The resulting *Ntab*MPO1-GFPox cell line exhibited punctuate signals suggesting that the GFP fusion of this enzyme was located in small organelles. To clarify the subcellular localization of MPO1, a peroxisome-targeted mCherry marker (PTS1-Cherry) [34] was transiently introduced into the *Ntab*MPO1-GFPox cell line. Since MPO1 was shown to be targeted to peroxisome in epidermal pavement cells of *Nicotiana benthamiana* [18], we transiently introduced by particle bombardment the peroxisomal mCherry marker PTS1-mCherry [34] into the background of the *Ntab*MPO1-GFP ox line expressing the enzyme as fusion with GFP. The tight colocalisation of the mCherry and the GFP signal (Fig 2) provides evidence for a correct localisation of the overexpressed MPO1.

**Fig 3:**
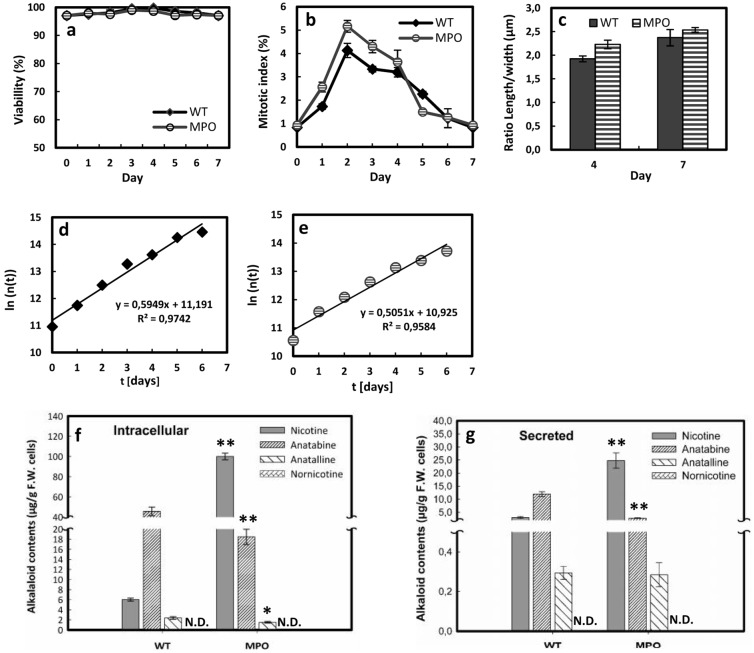
Phenotyping and alkaloid accumulation patterns for BY-2 cells overexpressing *Ntab*MPO1 compared to non-transformed BY-2 cells (WT). (**a)** cell viability (mean of n = 1000), **(b)** mitotic index (mean of n = 1000), **(c)** cell elongation as ratio of cell length over cell width at days 4 and 7 (mean of n = 500), **(d, e)** from the time course of cell density a cell cycle duration of 27.96 h for the non-transformed BY-2 cells **(d)** and of 32.9 h for BY-2 cells overexpressing *Ntab*MPO1 **(e)** can be inferred. All experimental data are derived from three independent experimental series; error bars = SE. **(f, g)** Alkaloid profiles measured after 3 days of culture in presence of 100 μM jasmonic acid either intracellularly **(f)** or secreted to the medium **(g)**. The levels of nornicotine were below detection limit (indicated by N.D.). Note the difference in scales between **(f)** and **(g)**. For the alkaloid measurement, mean and SE are shown from six independent experimental series. Significant differences to the non-transformed WT cells assessed by a Student’s t-test are indicated by * (*P* < 0.05) or ** (*P* < 0.01), respectively.

To detect potential side effects of the overexpression, the BY-2 MPO1-GFP ox line was monitored by quantitative phenotyping, using viability, mitotic index, cell elongation (in the sense of proportionality, assessed as ratio of cell length over width), and cycling time as parameters. Our phenotyping results did not detect any significant difference, neither in cell viability, nor with respect to mitotic index, or cell elongation (Fig 3a, 3b and 3c). Since mitotic index cannot only increase as consequence of stimulated proliferation, but also by delayed or arrested progression through mitosis, we quantified in addition the duration of the cell cycle. The doubling time can be inferred from the time course of cell density based on the model of exponential growth (Fig 3d and 3e). From this approach a longer cell cycle was inferred for the *Ntab*MPO1-GFP ox line compared to the non-transformed WT BY-2 cells. This delay in the *Ntab*MPO1 overexpressing line (from 28.0 h in the WT to 32.9 h in the overexpressor) was minor, however.

Despite the negligible impact of the overexpressed MPO on cellular physiology, the alkaloid profile was substantially changed after elicitation with jasmonic acid (Fig 3f and 3g). Preparatory experiments had shown that elicitations with 10 μM of jasmonic acid (JA) were only inducing a modest accumulation of alkaloids over the experimental period of 3 days (S2 Fig). However, by increasing the concentrations to 100 μM, the levels of alkaloids could be increased by a factor of 4 in case of nicotine, and more than 20 in case of anatabine (Fig 3f) compared to the treatment with 10 μM JA (S2 Fig). Under this optimised elicitation protocol, we observed that the intracellular content of nicotine was more than 15 times higher in the MPO overexpressor compared to the non-transformed wild type (Fig 3f). In contrast, the levels of anatabine (produced by the concurrent branch of the pathway) were decreased by around 3 times in the MPO overexpressor. Only residual levels of anatalline were measured, and the levels of nornicotine remained below detection limit. When the secreted alkaloids were analysed (Fig 3g), again a strong stimulation of nicotine and anatabine secretion was found in the MPO overexpressor and WT cells respectively, whereas the anatalline was comparable to the wild type. The general abundance of secreted alkaloids was around four times lower than those present within the cells indicating that only around 20% of the induced alkaloids were secreted.

### Overexpression of the nicotine demethylase *Ntab*CYP82E5*v*2 does not lead to nornicotine

Nornicotine is principally synthesised by enzymatic nicotine *N*-demethylation, catalysed by nicotine *N*-demethylase (NND), and mostly accumulates in the senescing leaves of specific tobacco species, so called converter species of the genus *Nicotiana*. However, low levels (2–5% of total alkaloid) of nornicotine can also be detected in green leaves of non-converter plants [15]. The enzyme underlying NND has been proposed to belong to the CYP82E clade of cytochrome P_450_ proteins. The residual NND activity in the green leaves found in the non-converter plant *N*. *tabacum* has been linked with *Ntab*CYP82E5*v*2 [15], [29], [43].

**Fig 4:**
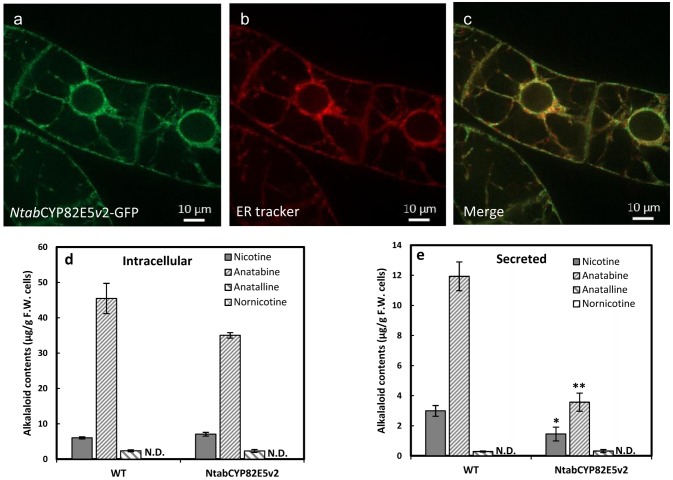
Localisation and metabolic impact of overexpressed *Ntab*CYP82E5*v*2-GFP. (**a**) *Ntab*CYP82E5*v*2-GFP signal, (**b**) rhodamine-conjugated ER-tracker, (**c**) merged signal of both channels showing the tight colocalisation of the nicotine demethylase *Ntab*CYP82E5*v*2 GFP with the endoplasmic reticulum. **(d, e)** Alkaloid profiles measured after 3 days of culture in presence of 100 μM jasmonic acid either intracellularly **(d)** or secreted to the medium **(e)**. The levels of nornicotine were below detection limit (indicated by N.D.). Note the difference in scales between **(d)** and **(e)** (the level of secreted alkaloids is in some case around tenfold lower). For the alkaloid measurement, mean and SE are shown from six independent experimental series. Significant differences to the non-transformed WT cells assessed by a Student’s t-test are indicated by * (*P* < 0.05) or ** (*P* < 0.01), respectively.

Therefore, we produced a transformed BY-2 cell line overexpressing the native enzyme, *Ntab*CYP82E5*v*2, originating from BY-2 in fusion with GFP under control of the CaMV 35S promoter. The GFP signal was organised in a reticulate pattern. To verify, whether this pattern was caused by a localisation in the endoplasmic reticulum, we used a rhodamine labelled version of the ER tracker and observed a tight colocalisation of both signals (Fig 4). We tested further, whether the overexpression of *Ntab*CYP82E5*v*2-GFP would alter cellular physiology. But neither cell viability, nor cell proportionality showed any significant differences between *Ntab*CYP82E5*v*2ox and the WT cells (S3 Fig). Only a minor increase in the progression of the mitotic index accompanied by a small acceleration of the cell cycle by around 5% could be noted.

Although the overexpressed nicotine demethylase *Ntab*CYP82E5*v*2 did not show any impact on cellular physiology, the alkaloid profile was substantially changed after elicitation with 100 μM of JA (Fig 4d and 4e): Although there were no significant differences in intracellular alkaloids between nicotine demethylase overexpressor and the non-transformed wild type (Fig 4d), significantly less nicotine and anatabine were secreted by nicotine demethylase overexpressor cells (Fig 4e). However, neither the intracellular, nor the secreted nicotine reached the levels found in the MPO1 overexpressor line (compare with Fig 3f and 3g). The effect was even more pronounced for anatabine, where the intracellular steady-state levels were almost the same in the nicotine demethylase overexpressor compared to the wild type, but nevertheless significantly lower amounts were secreted. Against the expectation, the overexpression of *Ntab*CYP82E5*v*2 did not yield any detectable nornicotine, neither in intracellular, nor secreted form (Fig 4d and 4e). Again, preparatory experiments were conducted with 10 μM of JA (S4 Fig) yielding a different pattern compared to 100 μM JA, and generally much lower induction of alkaloids. Both, intracellular and secreted nicotine and anatabine levels were significantly higher in the nicotine demethylase overexpressor compared to the wild type. One detail was interesting, however: The intracellular levels of nicotine and anatabine were already significantly elevated over those seen in the wild type at these low concentrations of the elicitor, whereas for 100 μM JA the wild type had the same (nicotine) or higher (anatabine) levels. This indicates a higher sensitivity of alkaloid synthesis in the nicotine demethylase overexpressor. Similar to the findings for the MPO1 overexpressor, the general abundance of secreted nicotine was about 4 times lower than those present within the cells and for anatabine around one order of magnitude lower than those present within the cells indicating that maximum around 10% of the induced alkaloids were secreted.

### Overexpression of a nicotine demethylase from a converter species leads to nornicotine

**Fig 5:**
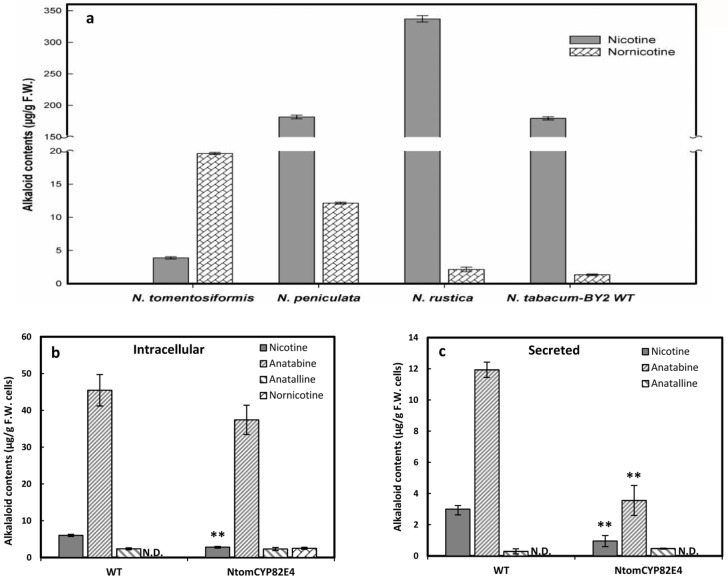
Nicotine and nornicotine contents in senescent leaves of different *Nicotiana* species and metabolic impact of overexpressed *Nom*CYP82E4-GFP. **(a)** The low ratio of nornicotine to nicotine in *N*. *tabacum* and the high level of nornicotine compared to nicotine in *N*. *tomentosiformis* are evident. Error bars represent SE from three independent experiments. **(b, c)** Alkaloid profiles measured after 3 days of culture in presence of 100 μM jasmonic acid either intracellularly **(b)** or secreted to the medium **(c)**. The levels of nornicotine below detection limit is indicated by non-detectable (N.D.). Note the difference in scales between **(b)** and **(c)** (the level of secreted alkaloids is in some case around tenfold lower). For the alkaloid measurement, mean and SE are shown from six independent experimental series. Significant differences to the non-transformed WT cells assessed by a Student’s t-test are indicated by * (*P* < 0.05) or ** (*P* < 0.01), respectively.

Since the overexpression of the native BY-2 nicotine demethylase *Ntab*CYP82E5*v*2 failed to generate nornicotine, we compared different genotypes of *Nicotiana* for their ability to accumulate nornicotine in senescent leaves (Fig 5a). Whereas the cultivar *N*. *tabacum* cv. Bright Yellow 2, the background for the BY-2 cell line, accumulated high levels of nicotine, the leaves were almost void of nornicotine. A similar pattern was observed for the species *N*. *rustica*. In contrast, *N*. *paniculata* and *N*. *tomentosiformis* were found to produce relatively high levels of nornicotine. In case of *N*. *tomentosiformis*, most of the nicotine was converted to nornicotine. This indicates that this species harbours a very efficient version of nicotine demethylase. For this reason, we cloned the nornicotine demethylase *Ntom*CYP82E4 from this converter species and overexpressed this gene in fusion with GFP in BY-2 cells to test, whether this cell line would be able to produce nornicotine. Similar to the native BY-2 nicotine demethylase *Ntab*CYP82E5*v*2, the *Ntom*CYP82E4-GFP fusion protein was localised in the endoplasmic reticulum and the physiology of this overexpressor line was completely normal with respect to viability and cell proportionality (S5 Fig). In addition, similar to the *Ntab*CYP82E5*v*2-GFP overexpressor, the cell cycle was accelerated slightly (by around 5%).

When the alkaloid accumulation in response to 100 μM of JA was investigated in this line expressing the nicotine demethylase from a converter species, we were able, for the first time, to observe nornicotine (Fig 5b). However, the level of nornicotine was still modest, and it seemed to be sequestered in the cells, since we could not detect any secreted nornicotine (Fig 5c). Compared to the non-transformed wild type, the nicotine demethylase overexpressor accumulated less nicotine. This reduced intracellular accumulation was accompanied by a significant decrease in the fraction of secreted nicotine and anatabine. When intracellular nornicotine was tested in response to 10 μM of JA (S5i Fig), both nornicotine and nicotine, were found to be already induced by this low concentration of the elicitor. In contrast, anatabine was not detected. This indicates a higher sensitivity of alkaloid synthesis for overexpression of the converter version nicotine demethylase overexpressor. But in contrast to the non-converter version of nicotine demethylase (S4 Fig), this increase in JA sensitivity seems to be limited to the nicotine branch of the pathway.

### Feeding of low concentrations of nicotine can release hidden potential for nornicotine accumulation in cell culture

**Fig 6:**
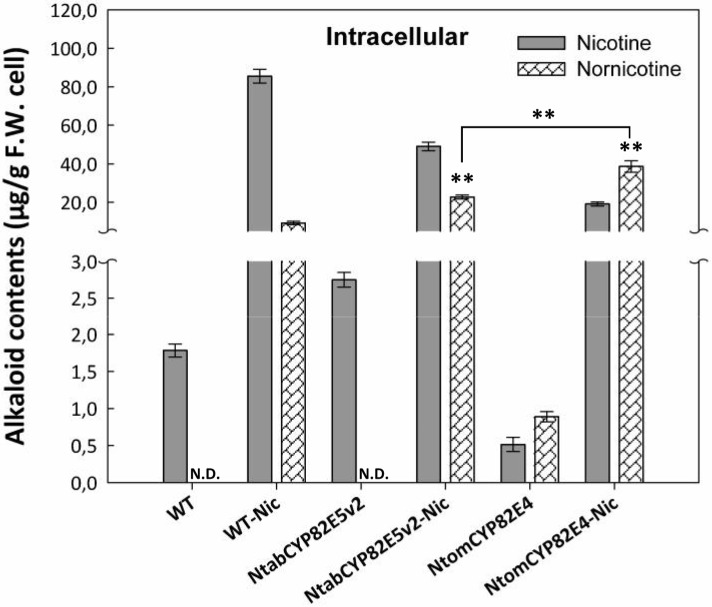
Alkaloid accumulation in non-transformed BY-2 cells (WT) compared to cells overexpressing *Ntab*CYP82E5*v*2 and *Ntom*CYP82E4, respectively after feeding with 15 μg^.^ml^-1^ of pure nicotine (Nic). The level of nornicotine below detection limit is indicated by non-detectable (N.D.). Error bars represent SE from 3 independent experimental series. Significant differences in nornicotine production to the WT treated with nicotine and also between two CYP overexpressing cell lines in a Student’s t-test are indicated by two (P < 0.01) asterisks.

Even with the nicotine demethylase version from the converter species *N*. *tomentosiformis*, the accumulation of nornicotine remained relatively modest (Fig 5b). The yield of this reaction might be limited by the availability of substrate for this overexpressed enzyme. Alternatively, it might be limited by regulatory factors that depend on the presence of the substrate. To address this question, we fed the precursor, nicotine (Fig 6). However, the quantity of nicotine was kept low (15 μg.ml^-1^). This should avoid that the added nicotine would simply overrun the accumulation of the nicotine produced by the cells themselves. This approach was efficient in stimulating the accumulation of intracellular nicotine and nornicotine even in non-transformed wild type cells. This increase of nicotine and nornicotine was about two orders of magnitude above the quantity of the added nicotine, i.e. it could not be explained in terms of addition or direct conversion of the added substrate. For the line overexpressing the GFP fusion of the non-converter nicotine methylase *Ntab*CYP82E5*v*2, the levels of nornicotine were doubled over the levels found in the nicotine-supplemented wild type, and this could be further doubled by feeding nicotine to the line overexpressing the converter nicotine methylase *Ntom*CYP82E4 in fusion with GFP. Here, the ratio of nicotine conversion was so high that the levels of nornicotine even exceeded those of nicotine, simulating the situation in the senescent leaves of this species (Fig 5). The observed significant increase of intracellular nornicotine in the treated samples might have been caused by gene activation of one of the endogenous nicotine demethylases (i.e. *NtabCYP82E5v2*, or *NtabCYP82E4*). However, the steady-state level of both transcripts was reduced after elicitation by jasmonic acid and was not stimulated by nicotine feeding, neither in presence, nor in the absence of jasmonic acid (S6 Fig). Also in the MPO1ox line, these transcripts were downregulated by jasmonic acid. Under control conditions, the CYPs genes were expressed in the MPO overexpressor at significantly lower levels as compared to wild type cells.

These experiments show that there exists a considerable potential for the accumulation of nornicotine even in the non-transformed wild type. This sleeping potential can be released by adding the precursor nicotine.

### Conditioned medium from BY-2 cells can stimulate alkaloid synthesis

In the natural context, nicotine is synthesised in the roots and transported into the leaves, where it is converted into nornicotine. In the aforementioned precursor feeding experiment, this situation was mimicked by confronting the *Ntom*CYP82E4-GFP overexpressor cells with nicotine. To develop this simulation of the situation *in planta* one step further, we asked, whether a similar effect might be produced by generating the precursor in a natural way, by secretion from elicited cells. This would provide a condition, where different metabolic modules can interact with each other. Similarly to the situation in the plant, cells with different metabolic capacity would be coupled together in a way that one cell type would produce and secret the compound which can act directly as precursor for the other cell type. Therefore, combination experiments were designed to investigate the regulatory or metabolic interaction of different cell types. To mimic the interaction found between the nicotine secreting cells of the root and the nicotine converting cells of the senescent leaf, we used conditioned medium from either non-transformed wild type or the MPO1 overexpressor which contains the precursor signal (secreted nicotine). Then, the *Ntom*CYP82E4-GFP overexpressor was cultivated in this conditioned medium.

**Fig 7:**
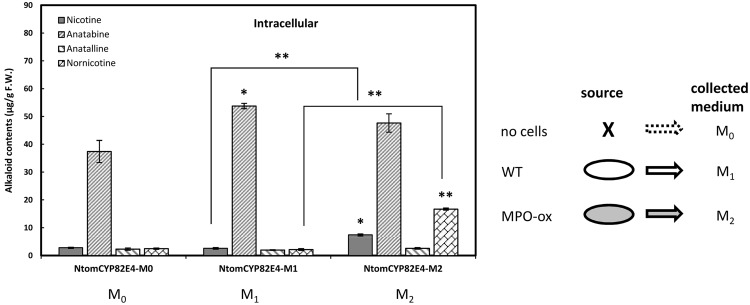
Profile of intracellular alkaloids accumulated in the *Ntom*CYP82E4 overexpressor in response to conditioned medium. Stimulation of intracellular alkaloid accumulation by conditioned medium collected from non-transformed wild type (M_1_) or cells overexpressing MPO1 (M_2_). Unconditioned medium (M_0_) was used as negative control. Alkaloid synthesis was elicited by 100 μM of jasmonic acid for three days. Error bars indicate SE, from three independent experimental series. Significant differences to the non-transformed WT cells assessed by a Student’s t-test are indicated by * (*P* < 0.05) or ** (*P* < 0.01), respectively.

For this experiment, medium of wild-type cells (Fig 7, M_1_) as control and *Ntab*MPO1-overexpressing cells (Fig 7, M_2_) was collected at day 3 and mixed with fresh medium separately. Thirty ml of these conditioned medium were used for subcultivation of cells overexpressing *Ntom*CYP82E4-GFP. After 3 days, the alkaloid level of *Ntom*CYP82E4 cells cultivated in the conditioned medium (M_1_ and M_2_) were analysed and compared with that of unconditioned medium (M_0_). The *Ntom*CYP82E4 cells were elicited with JA at a final concentration of 100 μM. We measured accumulation of nornicotine as our target compound and observed that it was stimulated significantly in *Ntom*CYP82E4 cells cultivated in conditioned medium M_2_ (Fig 7). We observed that the treatment with the conditioned medium M_1_ in *Ntom*CYP82E4 cells did not affect the intracellular nicotine and nornicotine production, but significantly stimulated the accumulation of intracellular anatabine (Fig 7). In contrast, when the conditioned medium was collected from the MPO1 overexpressor line (M_2_), the induction of intracellular nicotine was significantly enhanced over that observed for cultivation in unconditioned medium (M_0_). Strikingly, there was also a strong stimulation in the accumulation of nornicotine in response to conditioned medium M_2_ reaching around half of the levels achieved in the nicotine feeding experiment (Fig 6a). In contrast, conditioned medium M_1_, which contains very low amounts of secreted nicotine (about 100 ng.ml^-1^), was not efficient in stimulating nornicotine accumulation. Cultivation in the conditioned medium did not impair viability as verified by the Evans Blue dye exclusion test (S7 Fig).

### Co-cultivation of different cell types can efficiently release the metabolic potential for nornicotine synthesis

**Fig 8:**
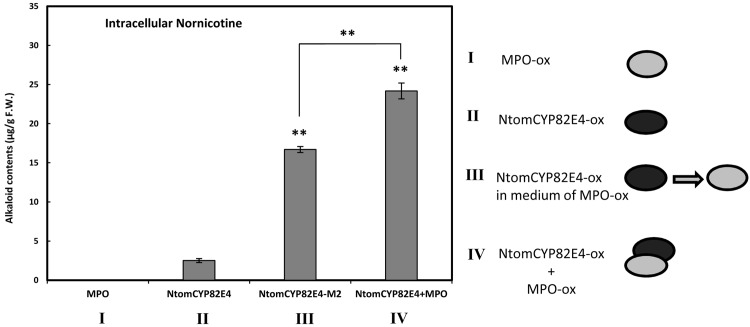
Nornicotine contents in BY-2 cells overexpressing *Ntab*MPO1 (I) and cells overexpressing *Ntom*CYP82E4 (II) compared to *Ntom*CYP82E4 cells cultivated in a mixture composed of medium of a three days old *Ntab*MPO1 cell line (M_2_) (III) and when co-cultivated together with *Ntab*MPO1 cells (IV). Error bars represent SE (n = 3). Significant differences to the *Ntom*CYP82E4 in a Student’s t-test are indicated by two asterisks (P < 0.01).

In the search for more efficient strategies to release the silent potential of the *Ntom*CYP82E4 overexpressor for nornicotine accumulation, we tested co-cultivation. In case that non-secreted factors present on the surface of one cell type would stimulate the metabolic potential of the recipient cell, this would not be mimicked by a transfer of conditioned medium. Since all overexpressor lines were generated using the same selection marker (KanR), such a co-cultivation strategy was feasible. We cultivated equal volumes of the *Ntab*MPO1 overexpressor and *Ntom*CYP82E4 overexpressors in the double volume of fresh medium and measured the nornicotine contents in cells after 3 days following elicitation with JA at a final concentration of 100 μM. Our results (Fig 8) show a strong increase in nornicotine accumulation, which exceeded the accumulation reached by cultivation in conditioned medium M_2_ from the MPO1 overexpressor (Fig 7, M_2_). This stimulation of nornicotine synthesis was accompanied by a concomitant decrease in steady-state levels of nicotine (S8 Fig). This indicates that non-soluble factors on the cell surface can stimulate the potential of the target cell (overexpressing *Ntom*CYP82E4) to convert nicotine to nornicotine.

## Discussion

By the use of molecular tools, the regulation of secondary metabolism has strongly accelerated the understanding of the biosynthetic pathways for natural products [44]. In order to achieve high yields for the final products, combinations of elicitor treatment, precursor feeding and metabolic engineering have been successfully employed. However, still many target molecules are not accessible, due to their chemical complexity or due to metabolic constraints. Still most plant secondary metabolites of commercial use such as the anti-cancer agents vinblastine and camptothecin are produced from extraction of plants, because better options are not available [6]. Metabolic engineering can be used in several ways to improve yields. To increase the carbon flux towards the desired product requires a detailed knowledge of the biosynthetic pathways involved. The most important step in metabolic engineering is to determine the rate-limiting enzymes as primary targets for genetic engineering. However, other factors such as regulatory aspects like feedback mechanisms and compartmentalisation must be considered as well [45], [46], [47]. In the current study, we used nicotine alkaloids exemplarily to demonstrate the importance of metabolic compartmentalisation into different cell types and tissue (roots versus leaves). Therefore, the main aim of the present work is to provide a condition in which metabolically different cell types could interact in order to increase the final product.

We were successful in generating two cell lines, where one line, *Ntab*MPO1, produces the precursor for the other line, *Ntom*CYP82E4. In all experiments, several transformant lines have been used with quantitatively the same results. We tested different strategies to connect these two metabolic modules. Our results suggest that although metabolic engineering was effective in case of *Ntab*MPO1 to improve the yield for the precursor nicotine, additional factors still may become limiting. For example, in our work, our target compound was nornicotine, but cells overexpressing the nicotine demethylase which were engineered to produce this target compound, failed to accumulate nornicotine. By feeding these cells with the precursor, nicotine, this bottleneck could be removed suggesting substrate availability and compartmentalisation as two limiting factors for the production of the final product by these engineered cells. Although precursor feeding is an alternative way to improve productivity, it is not cost-effective in most cases [48]. As alternative, we designed a strategy, where the precursor was supplied "in a natural way" by secretion from one metabolic module. This strategy to combine different metabolic modules was successful to release metabolic synergies that could not be produced by each of the modules alone.

### Nornicotine accumulation is controlled by nicotine

BY-2 cells overexpressing a key enzyme responsible for conversion of nicotine to nornicotine in *Nicotiana tabacum* (*Ntab*CYP82E5*v*2) were not able to produce nornicotine (Fig 4). However, the overexpression of a rate-limiting enzyme (*Ntom*CYP82E4) from the converter plant *Nicotiana tomentosiformis* leads to nornicotine accumulation although in minor amounts (Fig 5b). The low level of nornicotine production in BY-2 cells overexpressing nicotine demethylase might be caused by limited abundance of the precursor, nicotine. In fact, feeding a low concentration of nicotine as precursor led to a high accumulation of nornicotine in both CYP overexpressing cell lines as well as in BY-2 wild type cells (Fig 6). The potential for nornicotine biosynthesis by non-transformed BY-2 cells in presence of the precursor nicotine indicates that nornicotine accumulation is first controlled by nicotine and then by the abundance and enzymatic efficiency of the nicotine demethylase. However, alternative possibilities exist. For instance, the exogenously fed nicotine might be more readily available to the nornicotine demethylase because it enters through the cytosol. To address this, it would be necessary to discriminate endogenous and exogenous nicotine, for instance by using radioactively labelled nicotine. Irrespective of the intracellular compartmentalisation, one can conclude that the sleeping potential for nornicotine accumulation is released by adding nicotine.

### Nicotine acts as a signal not as a substrate in controlling nornicotine accumulation

A closer look on this precursor feeding experiment revealed, however, that the situation differs from simple substrate limitation. The concentrations of nicotine added were low (15 μg.ml^-1^), but caused an alkaloid production in both non-transformed and transformed BY-2 cells that was much higher (Fig 6). This is valid also for the synthesis of nicotine itself: When the non-transformed BY-2 cells were treated with this low concentration of nicotine, they produced more than 80 μg.g^-1^ fresh weight of cells nicotine and, in addition, a considerable amount of nornicotine compared to non-treated cells. A similar stimulation with even higher intracellular nornicotine accumulation could be generated in the overexpressor lines. This increase in accumulated nicotine alkaloids cannot be explained by a model, where the exogenous nicotine is simply imported and subsequently converted to nornicotine. The discrepancy between exogenous precursor and accumulated product rather suggest a regulatory role for nicotine. In other words, nicotine is not only acting as precursor but also as a signal able to stimulate the activity of the nicotine demethylase. To understand the mechanism behind this regulatory effect, we further measured the expression level of endogenous key genes involved in nornicotine biosynthesis (CYP82E4 and CYP82E5*v*2) in response to that low level of nicotine treatment in both non-transformed wild type and MPO overexpressor line (S6 Fig). Our results (S6 Fig) clearly show that the stimulation of nornicotine accumulation cannot be explained by transcriptional activation, but must either act at the level of compartmentalisation or enzymatic activity, i.e. by posttranslational mechanisms. In other words, nicotine could have an effect on capacity of cells to translate mRNA encoding nicotine demethylase enzymes. A further argument against a potential stimulation of nornicotine demethylase transcripts by nicotine is given by the observation that these transcripts are reduced in the MPO1 overexpressor, which accumulates and secretes more nicotine (S6 Fig).

### Nicotine is not the only signal controlling nornicotine accumulation

*Nicotiana tomentosiformis* is one of the ancestral species of *Nicotiana tabacum* with a high ability to convert nicotine to nornicotine linked with a specific version of nicotine demethylase, *Ntom*CYP82E4. The ability to accumulate nornicotine already in the green, non-senescent leaf is a characteristic feature that discriminates *N*. *tomentosiformis* from *N*. *tabacum* [30], [49], [50]. We were able to release this metabolic potential in the *Ntom*CYP82E4 overexpressor by feeding nicotine (Fig 5b). Although this strategy requires only small amounts of the precursor, because nicotine seems to act as a signal rather than as a substrate, this approach is still costly, which holds true for precursor feeding in general [48]. In the search for cost-effective alternatives, we tested a strategy, where the nicotine would come from the conditioned medium of other cells. Nicotine is the main precursor for nornicotine biosynthesis and its biosynthesis in *Nicotiana* species requires an oxidative deamination of N-methylputrescine, catalyzed by N-methyl putrescine oxidase (MPO1) [17], [25]. The intracellular and secreted nicotine in tobacco BY-2 cells is very low compared to anatabine because MPO1 is hardly expressed in these cells [25]. We therefore used a transgenic line overexpressing MPO1, because these cells were able, after elicitation to produce and secret nicotine in a relatively high amount (Fig 3f and 3g).

This strategy was successful: Using the medium from elicited BY-2 cells overexpressing *Ntab*MPO1, we were able to get an eightfold increase of nornicotine accumulation in the *Ntom*CYP82E4 overexpressor (Fig 7). We wondered, whether this stimulation was caused by the secreted nicotine in the conditioned medium M_2_. However, when the concentration of nicotine in this conditioned medium is estimated based on the data shown in Fig 3g, it is found to be very low (only 500 ng.ml^-1^ compared to the 15 μg.ml^-1^ used in the nicotine feeding experiment). Thus, although the secreted nicotine concentration in the conditioned medium M_2_ is around 30 times lower than the nicotine used in the feeding experiment (Fig 6a), the nornicotine accumulation induced by the conditioned medium M_2_ reached half of the values achieved by feeding of a much higher concentration of exogenous nicotine. This means that the stimulation of nornicotine accumulation by the conditioned medium M_2_ cannot be explained by the traces of nicotine present in this medium, but must be triggered by a different positive regulator. Since the viability of cells cultivated in conditioned medium M_1_ or M_2_ was completely normal, precluding a scenario that nornicotine accumulation was triggered in consequence of senescence as response to stress (S7 Fig).

Co-cultivation of *Ntab*MPO1 and *Ntom*CYP82E4 transgenic lines together was another logical alternative and additional method to provide an environment for cell interaction. Co-culture of root and shoot has beed used previously to improve production of tropane alkaloids in *Atropa belladonna* and a *Duboisia leichhardtii* × *D*. *myoporoides* hybrid plants [51]. The significant increase in nornicotine production by *Ntom*CYP82E4 when co-cultivated with *Ntab*MPO1 cells demonstrated that the physical presence of cells was superior to just feeding the conditioned medium collected from the same cells (Fig 8). At the current state, this result is surprising, and there are four scenarios that should be pursued in the future: Non-diffusible signals of the extracellular matrix might be involved in metabolic regulation, and efficient exchange of these signals would require physical contact of the two cell types. Alternatively, there might be reciprocal signalling from the cells accumulating nornicotine (*Ntom*CYP82E4) on those that produce nicotine (*Ntab*MPO1), and this reciprocal signalling stimulates the production of the positive regulator secreted by the precursor cells (*Ntab*MPO1). There might be also an unstable signal produced by *Ntab*MPO cells stimulating production of nornicotine by *Ntom*CYP82E4 cells which is already degraded in the condition medium M2. The last scenario considers the difference in timing: In the M_2_ conditioned medium experiment, the *Ntom*CYP82E4 cells are exposed to the unknown factor secreted by the *Ntab*MPO cells from the very beginning. In the co-cultivation experiment, the *Ntab*MPO cells have first to accumulate and secrete this factor, such that the *Ntom*CYP82E4 cells will first proliferate in the absence or under only low levels of this factor. Only with time, the *Ntom*CYP82E4 cells are exposed to the higher levels present in the M_2_ conditioned medium. The conditioned medium might not only contain compounds that stimulate the nornicotine synthesis, but also compounds that slow down cell division. This scenario is also indicated by the finding that the cell cycle in the *Ntab*MPO1 line is a bit slower than in the wild type, which might be due to such secreted factors accumulating in the medium (Fig 3e).

## Outlook

Our present work suggests a strategy to technically mimic the cooperation of different cell types in a plant tissue in order to release hidden metabolic potentials and obtain valuable secondary metabolites that otherwise would not be produced in cell culture. Compared to alternative strategies, such as multiplex transformation, the combination strategy used in the current work has the advantage that it can be easily extended and adapted in a modular manner leading to a high degree of versatility. Future work will be dedicated to identify the unknown regulatory factors in the conditioned medium. Moreover, the strategy might be further improved by integrating temporal dynamics to address cell proliferation and metabolic activity differently. For this purpose, microfluidic strategies [52] will be used as platform to integrate different plant-cell based metabolic modules.

## Supporting Information

S1 Fig High-performance liquid chromatography (HPLC) profile of the reference mixture of nicotinic alkaloids (**a**). Diode array detection (HPLC-DAD; 260 nm) chromatogram of pure standards nornicotine (**b**), anabasine (**c**), anatabine (**d**), anatalline (two isomeric forms) (**e**), and nicotine (**f**).
(TIF)

S2 Fig Alkaloid profiles measured in non-transformed BY-2 cells (WT) and cells overexpressing *Ntab*MPO1 (MPO) after 3 days of culture in presence of 10 μM jasmonic acid either intracellularly (a) or secreted to the medium (b). The levels of nornicotine and in some case anatabine were below detection limit (indicated by N.D.). Note the difference in scales between **(a)** and **(b)**. For the alkaloid measurement, mean and SE are shown from six independent experimental series. Significant differences to the non-transformed WT cells assessed by a Student’s t-test are indicated by * (*P* < 0.05) or ** (*P* < 0.01), respectively.
(TIF)

S3 Fig Phenotyping of BY-2 cells overexpressing *Ntab*CYP82E5*v*2 compared to non-transformed BY-2 cells (WT). (**a)** Cell viability (mean of n = 1000), **(b)** Mitotic index (mean of n = 1000), **(c)** Cell elongation as ratio of cell length over cell width in day 4 and 7 (mean of n = 500), **(d, e)** From the time course of cell density a cell cycle duration of 28.0 h for the non-transformed BY-2 cells **(d)** and of 26.4 h for BY-2 cells overexpressing *Ntab*CYP82E5*v*2 can be inferred. All experimental data are derived from three independent experimental series; error bars = SE.
(TIF)

S4 Fig Alkaloid profiles measured in non-transformed BY-2 cells (WT) and cells overexpressing *Ntab*CYP82E5*v*2 after 3 days of culture in presence of 10 μM jasmonic acid either intracellularly (a) or secreted to the medium (b). The levels of nornicotine and in some cases anatabine and anatalline were below detection limit (indicated by N.D.). Note the difference in scales between **(a)** and **(b)**. For the alkaloid measurement, mean and SE are shown from six independent experimental series. Significant differences to the non transformed WT cells assessed by a Student’s t-test are indicated by * (*P* < 0.05) or ** (*P* < 0.01), respectively.
(TIF)

S5 Fig Localisation, physiological, and metabolic impact of overexpressed *Ntom*CYP82E4-GFP. (**a**) *Ntom*CYP82E4-GFP signal, (**b**) Rhodamine-conjugated ER-tracker, (**c**) merged signal of both channels showing the tight colocalisation of the nicotine demethylase *Ntom*CYP82E4-GFP with the endoplasmic reticulum. (**d)** Cell viability (mean of n = 1000), **(e)** Mitotic index (mean of n = 1000), **(f)** Cell elongation as ratio of cell length over cell width in day 4 and 7 (mean of n = 500), **(g, h)** From the time course of cell density a cell cycle duration of 27.2 h for the non-transformed BY-2 cells **(g)** and of 25.2 h for BY-2 cells overexpressing *Ntom*CYP82E4 can be inferred. All experimental data are derived from three independent experimental series; error bars = SE. **(i)** Intracellular alkaloid profiles measured in non-transformed BY-2 cells (WT) and cells overexpressing *Ntom*CYP82E4 after 3 days of culture in presence of 10 μM jasmonic acid. For the alkaloid measurement, mean and SE are shown from six independent experimental series. Non-detectable alkaloids are indicated by (N.D.). Significant differences to the non-transformed WT cells assessed by a Student’s t-test are indicated by * (*P* < 0.05) or ** (*P* < 0.01), respectively.
(TIF)

S6 Fig Steady state levels of the *Ntab*CYP82E4 (a) and the *Ntab*CYP82E5*v*2 (b) transcripts in non-transformed BY-2 cells (WT) and MPO overexpressor line elicited with jasmonic acid at a final concentration of 10 μM and 100 μM and without elicitation as well as WT cells treated with 15 μg ml^-1^ nicotine and jasmonic acid at the same time and treated with nicotine alone normalized to L25 ribosomal protein and elongation factor 1α (EF-1 α) as internal standards. Error bars indicate SE from three independent experimental series. Significant differences of *Ntab*MPO1 (elicited and non-elicited) to the WT (elicited and non-elicited) assessed by a Student’s t-test are indicated by * (*P* < 0.05) or ** (*P* < 0.01), respectively.
(TIF)

S7 Fig Viability of BY-2 cells overexpressing *Ntom*CYP82E4 elicited with jasmonic acid at a final concentration of 10 μM and 100 μM, and when cultivated in conditioned medium of non-transformed (M_1_) and *Ntab*MPO1 overexpressing cells (M_2_) (n = 500). Data are derived from three independent experimental series. Error bars represent SE.
(TIF)

S8 Fig Nicotine and nornicotine contents in BY-2 cells overexpressing *Ntom*CYP82E4 co-cultured together with *Ntab*MPO1 compared to *Ntab*MPO1 and *Ntom*CYP82E4 cell lines. The nornicotine level in MPO line was below detection limit (indicated by N.D.) Error bars represent SE (n = 3). Significant differences to the *Ntom*CYP82E4 and *Ntab*MPO1 assessed by a Student’s t-test are indicated by * (*P* < 0.05) or ** (*P* < 0.01), respectively.
(TIF)
