# Supplementary figures and images for: Combination of Plant Metabolic Modules Yields Synthetic Synergies
Source: PLoS One. 2017 Jan 12;12(1):e0169778. doi: 10.1371/journal.pone.0169778 (PMC5231347; doi:10.1371/journal.pone.0169778)

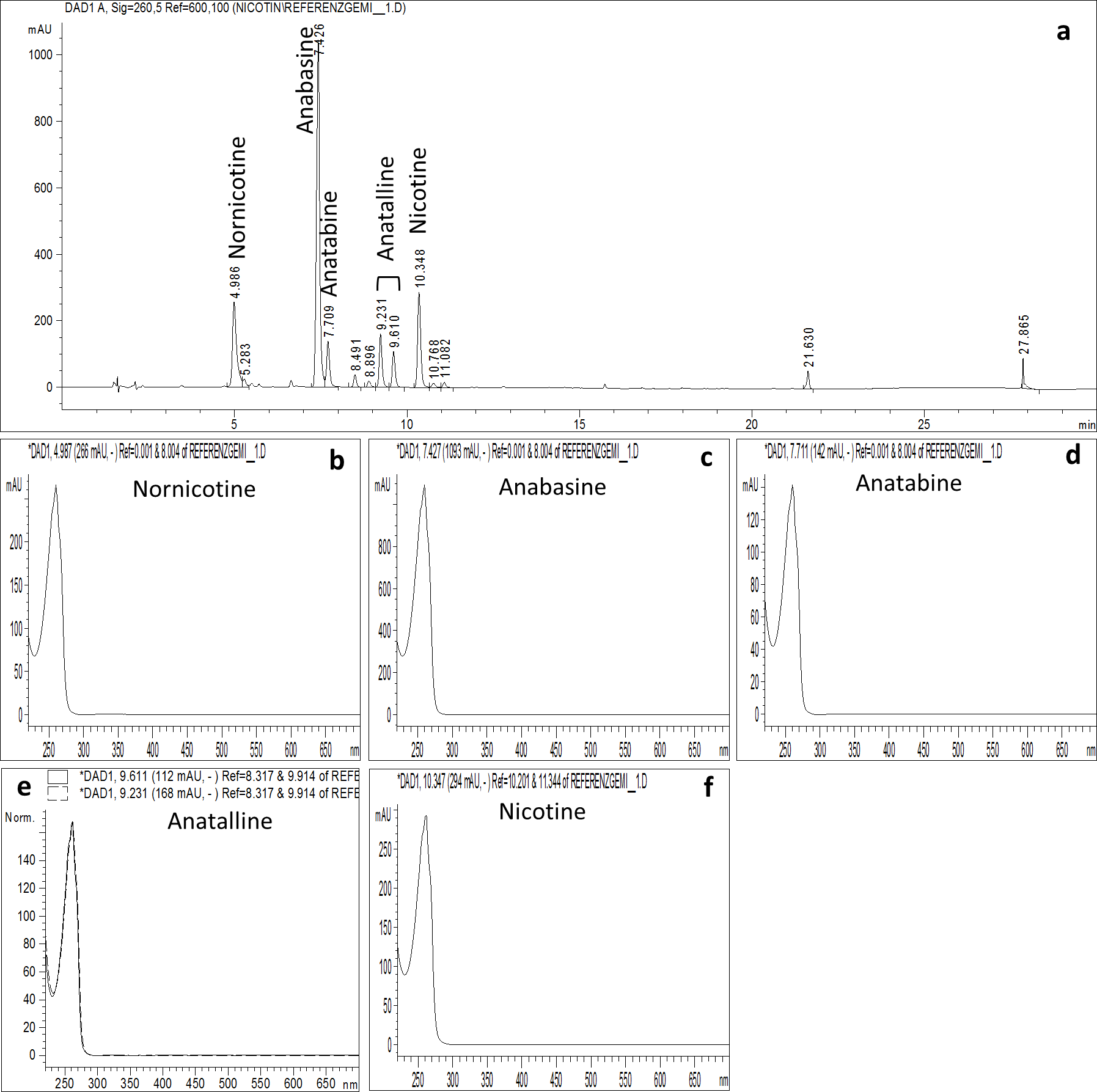

Supplement: S1 Fig — High-performance liquid chromatography (HPLC) profile of the reference mixture of nicotinic alkaloids (a). Diode array detection (HPLC-DAD; 260 nm) chromatogram of pure standards nornicotine (b), anabasine (c), anatabine (d), anatalline (two isomeric forms) (e), and nicotine (f). (TIF) [file pone.0169778.s001.tif]

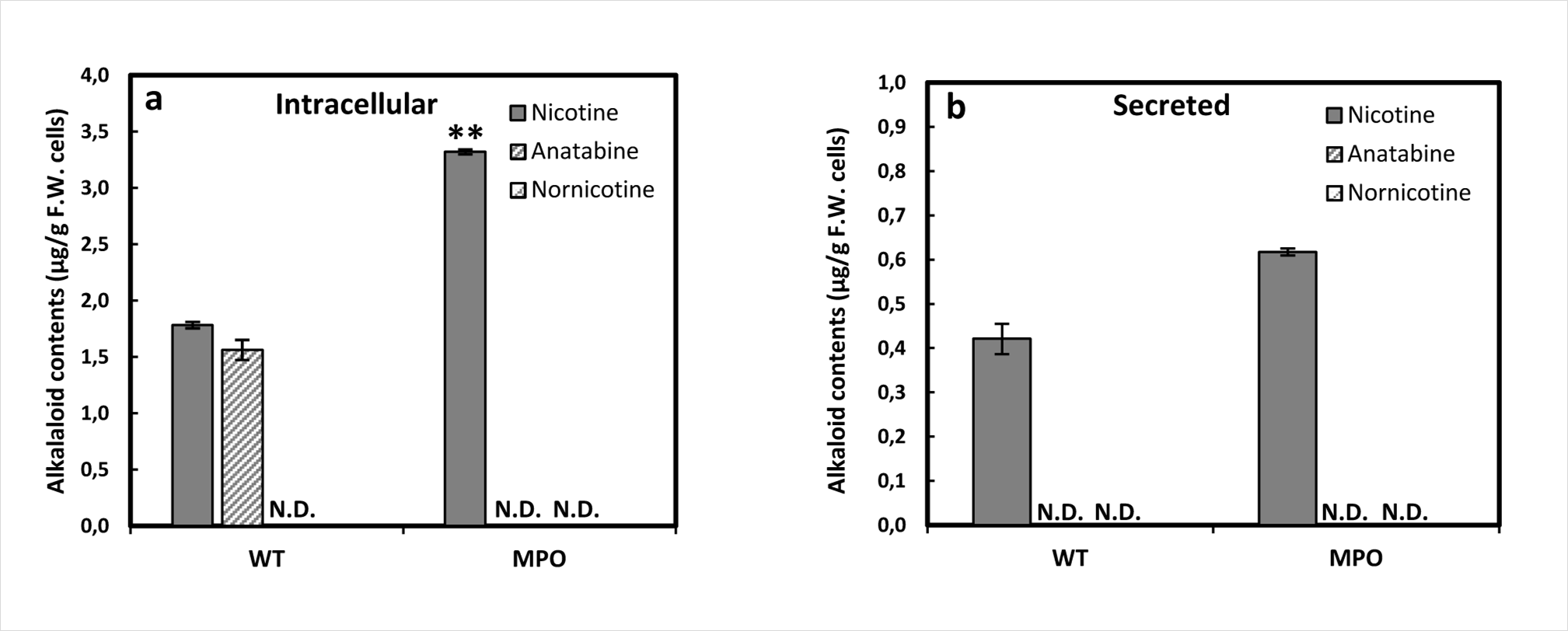

Supplement: S2 Fig — The levels of nornicotine and in some case anatabine were below detection limit (indicated by N.D.). Note the difference in scales between (a) and (b). For the alkaloid measurement, mean and SE are shown from six independent experimental series. Significant differences to the non-transformed WT cells assessed by a Student’s t-test are indicated by * (P < 0.05) or ** (P < 0.01), respectively. (TIF) [file pone.0169778.s002.tif]

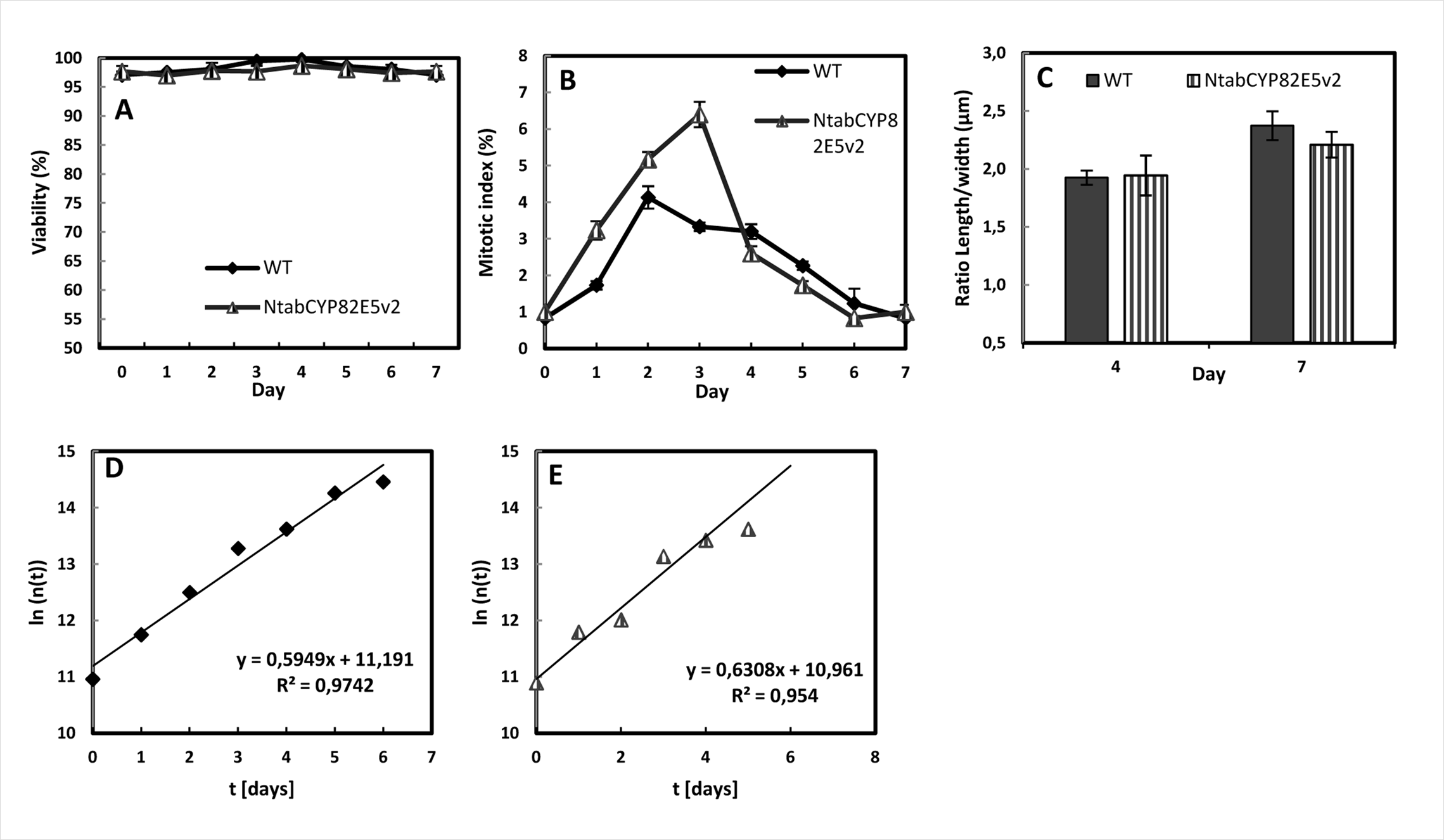

Supplement: S3 Fig — (a) Cell viability (mean of n = 1000), (b) Mitotic index (mean of n = 1000), (c) Cell elongation as ratio of cell length over cell width in day 4 and 7 (mean of n = 500), (d, e) From the time course of cell density a cell cycle duration of 28.0 h for the non-transformed BY-2 cells (d) and of 26.4 h for BY-2 cells overexpressing NtabCYP82E5v2 can be inferred. All experimental data are derived from three independent experimental series; error bars = SE. (TIF) [file pone.0169778.s003.tif]

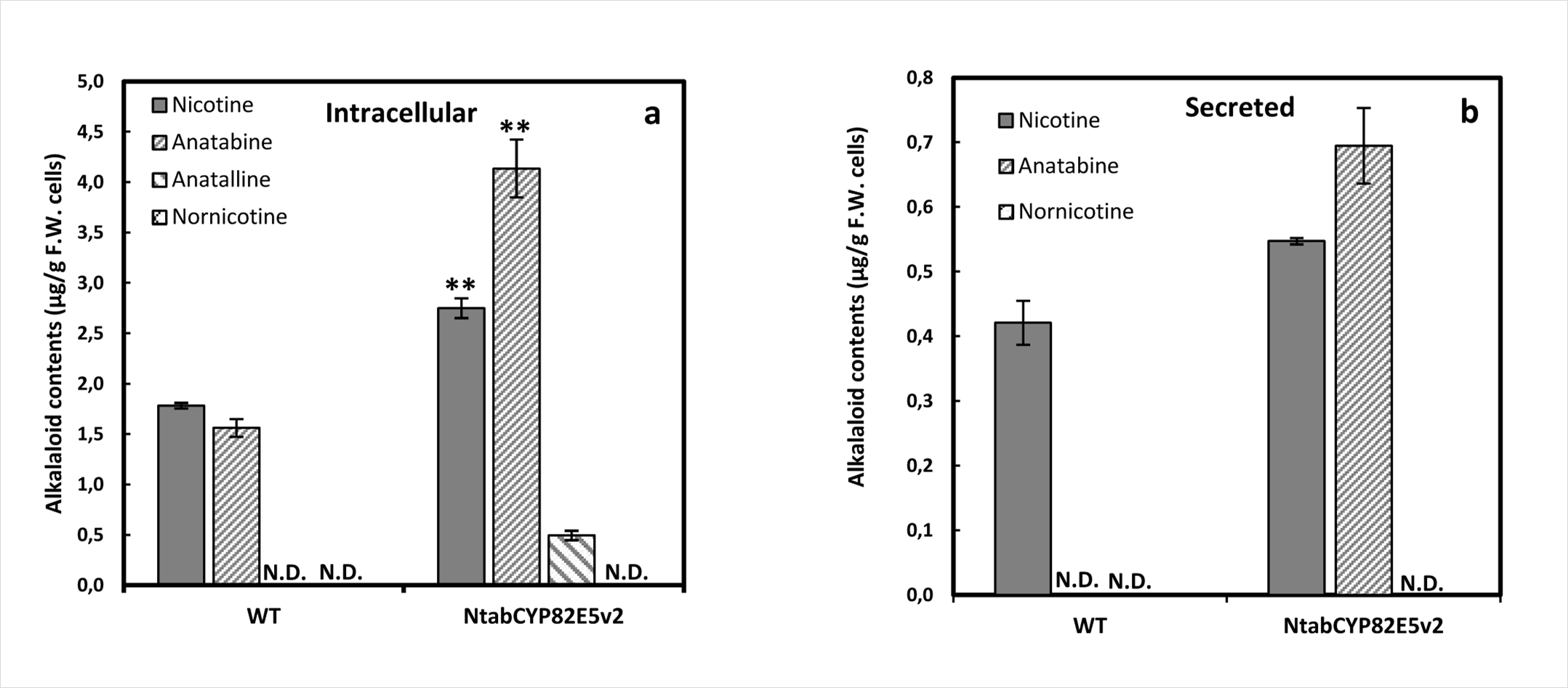

Supplement: S4 Fig — The levels of nornicotine and in some cases anatabine and anatalline were below detection limit (indicated by N.D.). Note the difference in scales between (a) and (b). For the alkaloid measurement, mean and SE are shown from six independent experimental series. Significant differences to the non transformed WT cells assessed by a Student’s t-test are indicated by * (P < 0.05) or ** (P < 0.01), respectively. (TIF) [file pone.0169778.s004.tif]

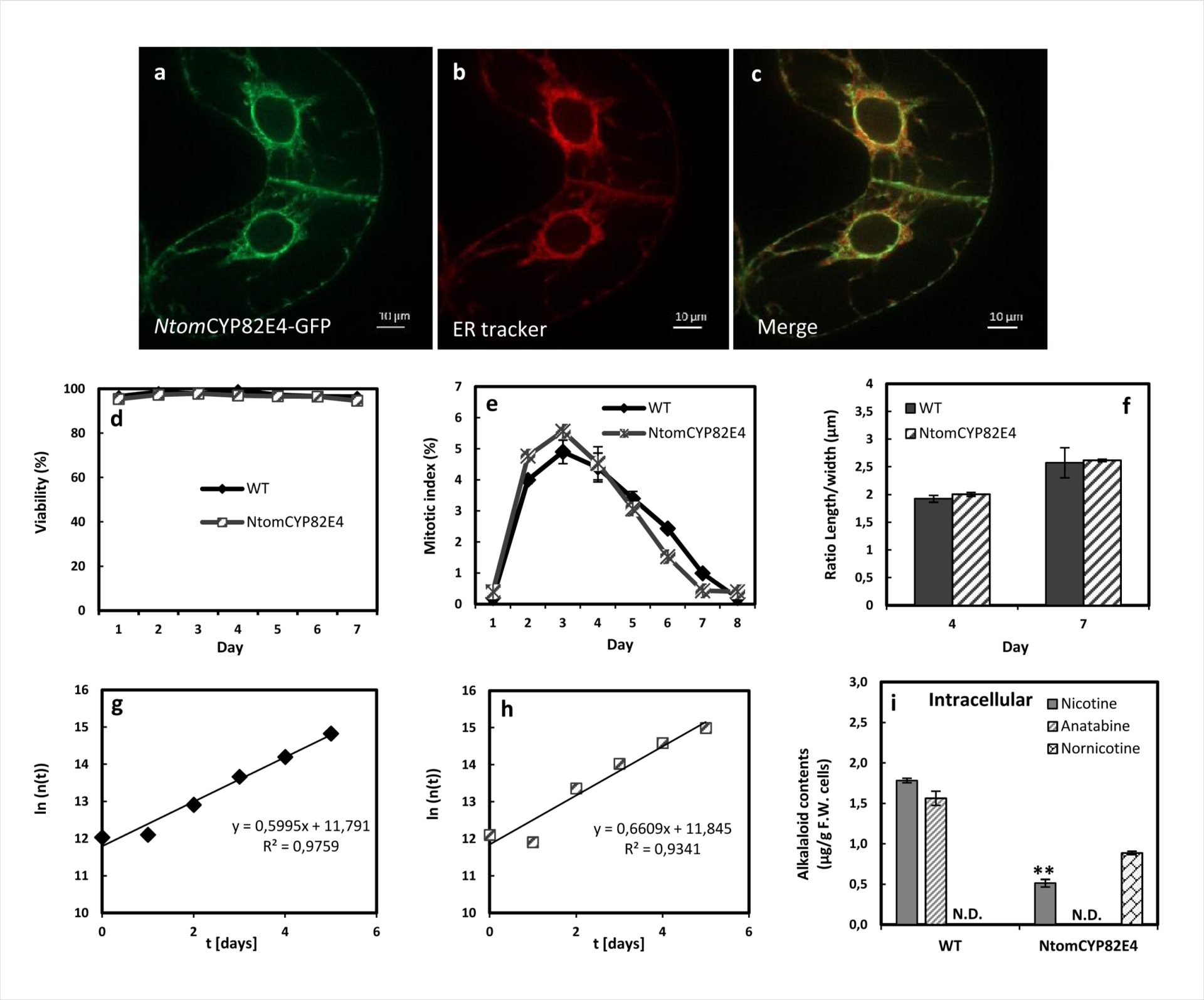

Supplement: S5 Fig — (a) NtomCYP82E4-GFP signal, (b) Rhodamine-conjugated ER-tracker, (c) merged signal of both channels showing the tight colocalisation of the nicotine demethylase NtomCYP82E4-GFP with the endoplasmic reticulum. (d) Cell viability (mean of n = 1000), (e) Mitotic index (mean of n = 1000), (f) Cell elongation as ratio of cell length over cell width in day 4 and 7 (mean of n = 500), (g, h) From the time course of cell density a cell cycle duration of 27.2 h for the non-transformed BY-2 cells (g) and of 25.2 h for BY-2 cells overexpressing NtomCYP82E4 can be inferred. All experimental data are derived from three independent experimental series; error bars = SE. (i) Intracellular alkaloid profiles measured in non-transformed BY-2 cells (WT) and cells overexpressing NtomCYP82E4 after 3 days of culture in presence of 10 μM jasmonic acid. For the alkaloid measurement, mean and SE are shown from six independent experimental series. Non-detectable alkaloids are indicated by (N.D.). Significant differences to the non-transformed WT cells assessed by a Student’s t-test are indicated by * (P < 0.05) or ** (P < 0.01), respectively. (TIF) [file pone.0169778.s005.tif]

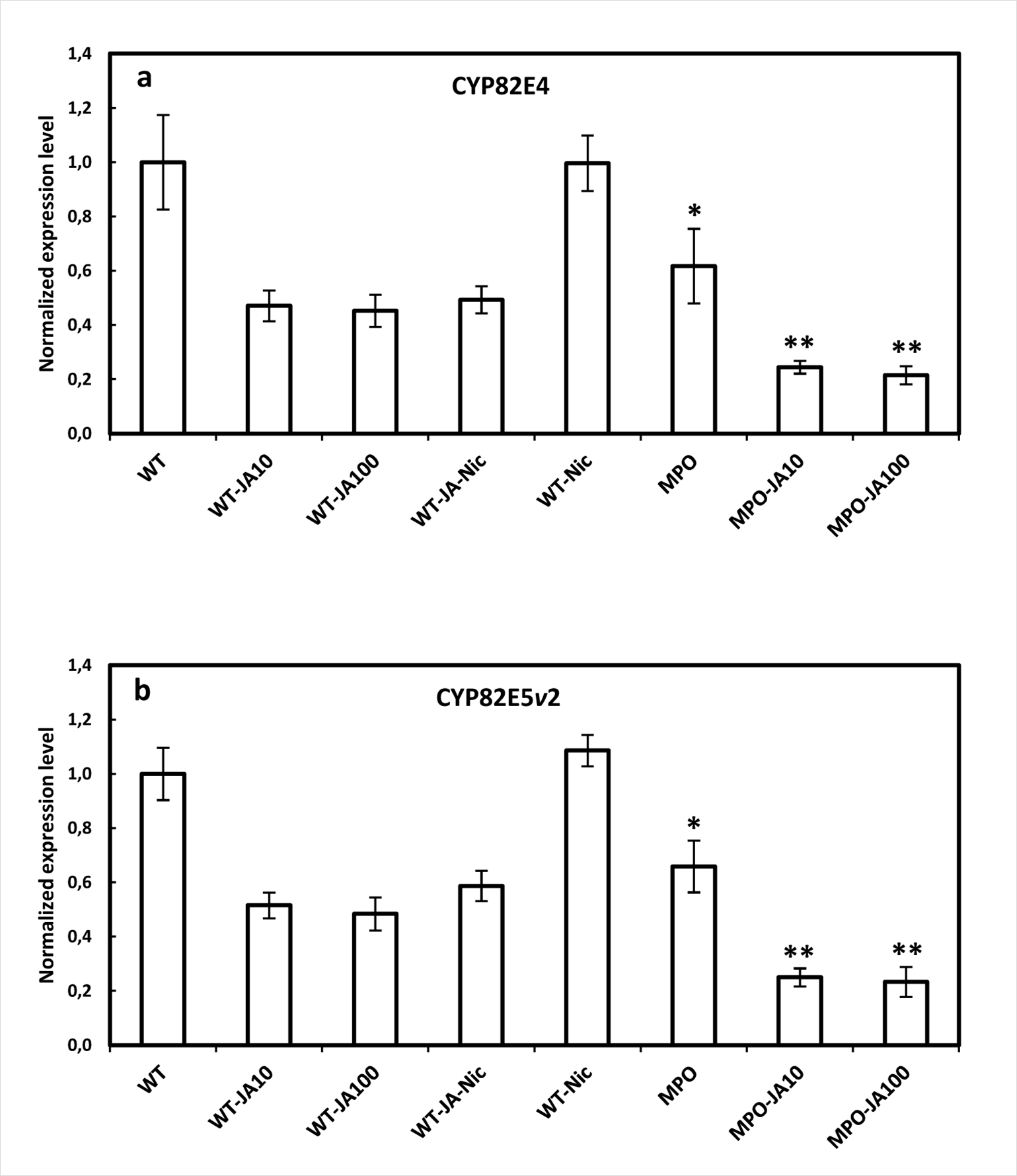

Supplement: S6 Fig — Error bars indicate SE from three independent experimental series. Significant differences of NtabMPO1 (elicited and non-elicited) to the WT (elicited and non-elicited) assessed by a Student’s t-test are indicated by * (P < 0.05) or ** (P < 0.01), respectively. (TIF) [file pone.0169778.s006.tif]

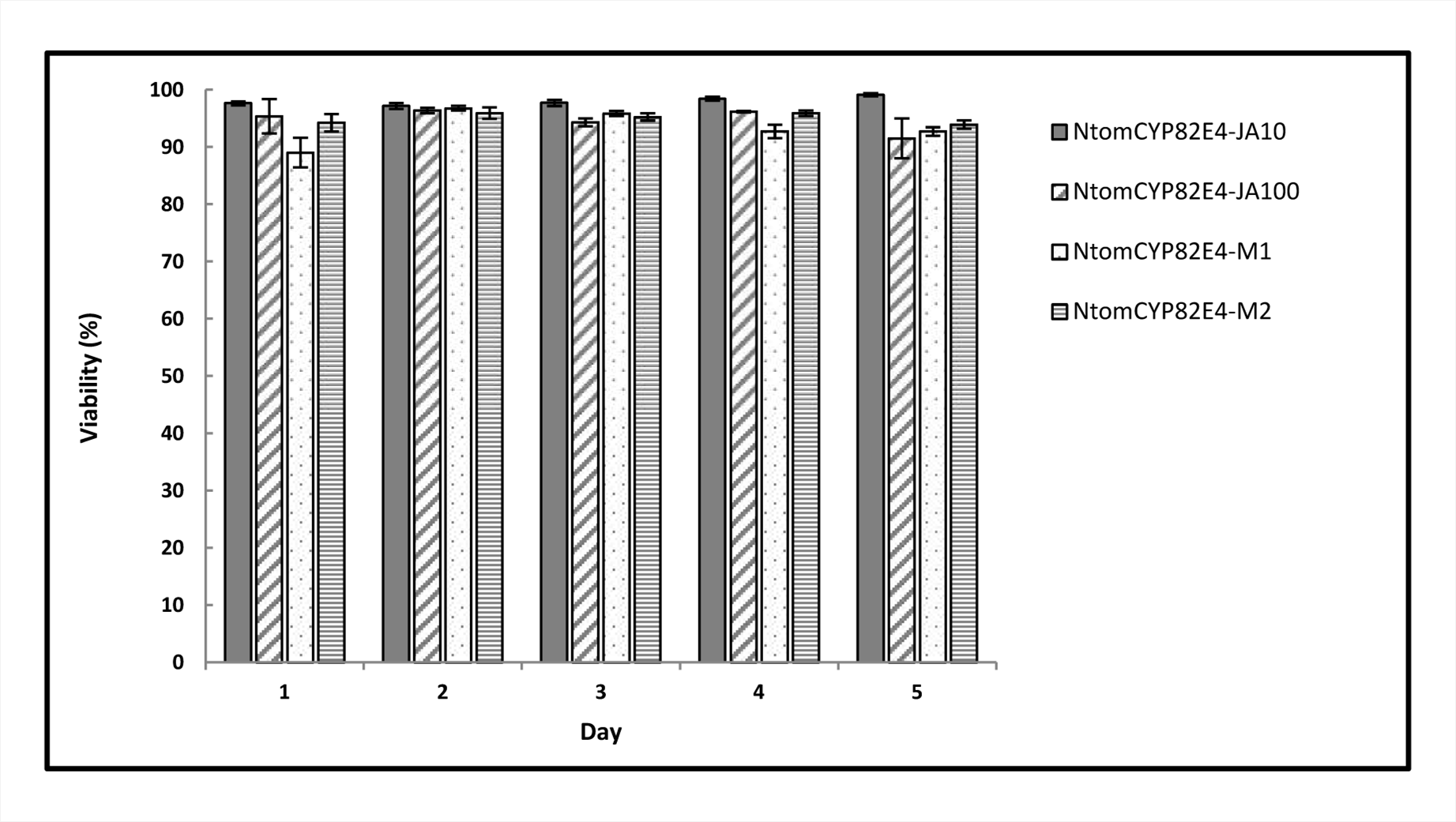

Supplement: S7 Fig — Data are derived from three independent experimental series. Error bars represent SE. (TIF) [file pone.0169778.s007.tif]

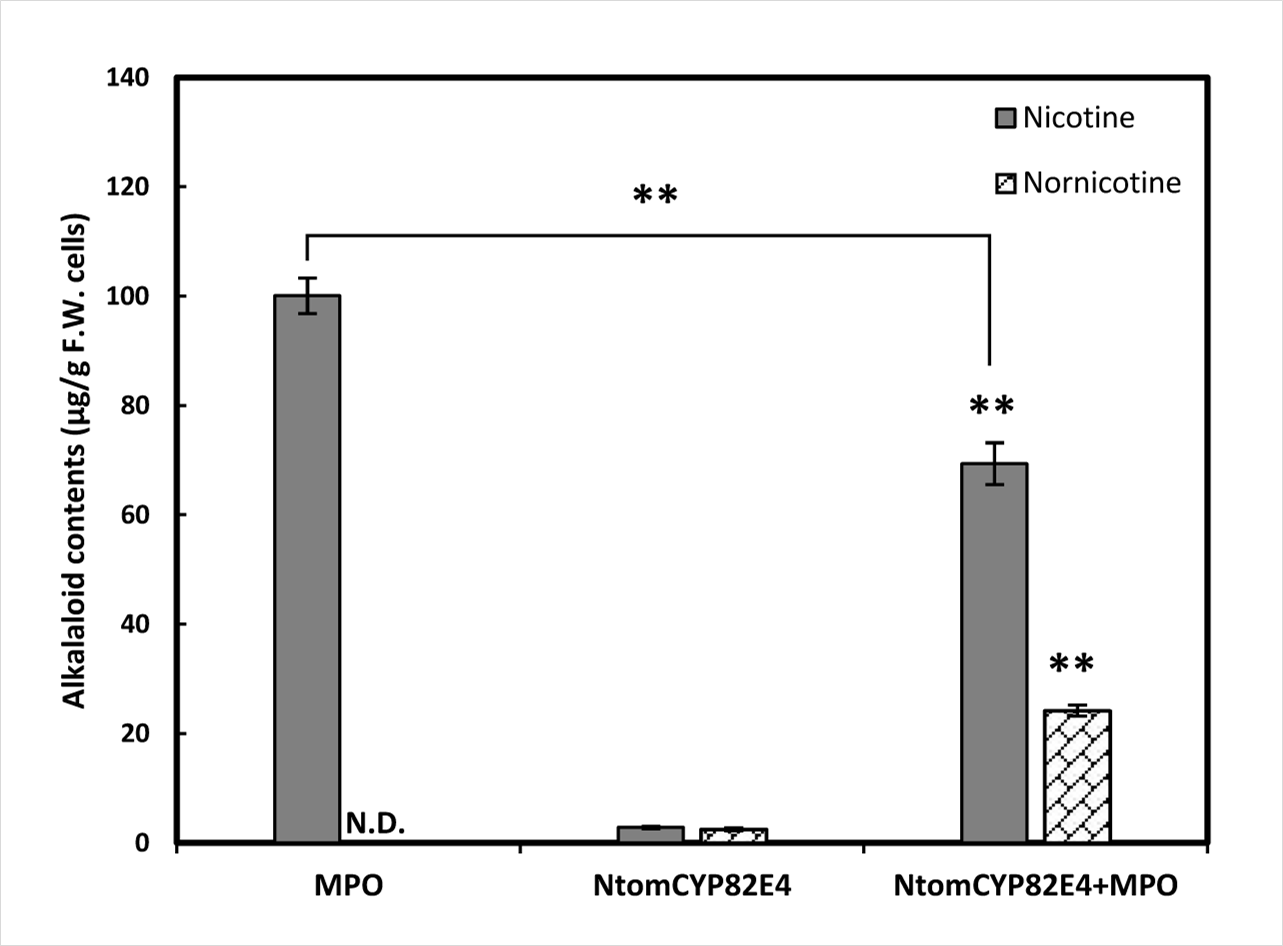

Supplement: S8 Fig — The nornicotine level in MPO line was below detection limit (indicated by N.D.) Error bars represent SE (n = 3). Significant differences to the NtomCYP82E4 and NtabMPO1 assessed by a Student’s t-test are indicated by * (P < 0.05) or ** (P < 0.01), respectively. (TIF) [file pone.0169778.s008.tif]
